# COVID-19 in hematopoietic stem cell transplant recipients during three years of the pandemic: a multicenter study in Brazil

**DOI:** 10.1590/S1678-9946202466017

**Published:** 2024-03-18

**Authors:** Bruno Azevedo Randi, Hermes Ryoiti Higashino, Vinícius Ponzio da Silva, Matias Chiarastelli Salomão, Antonio Carlos Campos Pignatari, Edson Abdala, Fabiana Vasques, Celso Arrais Rodrigues da Silva, Roberto Luiz da Silva, Carolina dos Santos Lazari, José Eduardo Levi, Erick Menezes Xavier, Marina Farrel Côrtes, Alessandra Luna-Muschi, Vanderson Rocha, Silvia Figueiredo Costa

**Affiliations:** 1 Universidade de São Paulo, Faculdade de Medicina, Hospital das Clínicas, Departamento de Moléstias Infecciosas e Parasitárias, São Paulo, São Paulo, Brazil; 2 Hospital 9 de Julho, Dasa, São Paulo, São Paulo, Brazil; 3 Universidade Federal de São Paulo, Escola Paulista de Medicina, São Paulo, São Paulo, Brazil; 4 Universidade de São Paulo, Faculdade de Medicina, Hospital das Clínicas, Divisão de Laboratório Central, São Paulo, São Paulo, São Paulo, Brazil; 5 Dasa, São Paulo, São Paulo, Brazil; 6 Universidade de São Paulo, Faculdade de Medicina, Hospital das Clínicas, Departamento de Hematologia, Hemoterapia e Terapia Celular, São Paulo, São Paulo, Brazil; 7 Universidade de São Paulo, Faculdade de Medicina, Laboratório de Investigação Médica em Protozoologia, Bacteriologia e Resistência Antimicrobiana (LIM-49), São Paulo, São Paulo, Brazil; 8 Universidade de São Paulo, Faculdade de Medicina, Instituto de Medicina Tropical de São Paulo, São Paulo, São Paulo, Brazil

**Keywords:** COVID-19, SARS-CoV-2, Hematopoietic stem cell transplantation

## Abstract

Hematopoietic stem cell transplant (HSCT) recipients are at -increased risk for severe COVID-19. The aim of this study was to evaluate the burden of COVID-19 in a cohort of HSCT recipients. This retrospective study evaluated a cohort of adult hospitalized HSCT recipients diagnosed with COVID-19 in two large hospitals in São Paulo, Brazil post-HSCT, from January 2020 to June 2022. The primary outcome was all-cause mortality. Of 49 cases, 63.2% were male with a median age of 47 years. Allogeneic-HSCT (51.2%) and autologous-HSCT (48.9%) patients were included. The median time from HSCT to COVID-19 diagnosis was 398 days (IQR: 1211-134), with 22 (44.8%) cases occurring within 12 months of transplantation. Most cases occurred during the first year of the pandemic, in non-vaccinated patients (n=35; 71.4%). Most patients developed severe (24.4%) or critical (40.8%) disease; 67.3% received some medication for COVID-19, primarily corticosteroids (53.0%). The probable invasive aspergillosis prevalence was 10.2%. All-cause mortality was 40.8%, 51.4% in non-vaccinated patients and 14.2% in patients who received at least one dose of the vaccine. In the multiple regression analyses, the variables mechanical ventilation (OR: 101.01; 95% CI: 8.205 – 1,242.93; *p* = 0.003) and chest CT involvement at diagnosis ≥50% (OR: 26.61; 95% CI: 1.06 – 664.26; *p* = 0.04) remained associated with all-cause mortality. Thus, HSCT recipients with COVID-19 experienced high mortality, highlighting the need for full vaccination and infection prevention measures.

## INTRODUCTION

Immunocompromised patients, such as hematopoietic stem cell transplant (HSCT) recipients, are at a higher risk for severe COVID-19^1^. As a result, many transplant - centers reduced the number of HSCT procedures during the initial months of the pandemic. This approach was in line with guidelines established by the European Society for Blood and Marrow Transplantation (EBMT), which recommended delaying transplantation for chronic non-malignant diseases whenever possible^2^.

A limited number of studies have reported COVID-19 outcomes in the HSCT setting^3-6^. The objective of this study was to describe COVID-19 burden in a cohort of HSCT recipients who were hospitalized in São Paulo, Brazil during three years of the pandemic.

## MATERIALS AND METHODS

### Study design

This was a retrospective observational study carried out in two large hospitals in the city of São Paulo, Brazil. The centers that contributed to this study were Hospital das Clinicas da Faculdade de Medicina da Universidade de Sao Paulo (HC-FMUSP) and Hospital 9 de Julho. The first one is the largest public university hospital in Latin America and has a total of 920 beds, including 12 dedicated to the Bone Marrow Transplantation (BMT) unit. The second center, Hospital 9 de Julho, is a private facility that has 470 beds including 14 in the BMT unit. For greater clarity in the presentation of results, these centers were called Hospital 1 and Hospital 2, respectively.

All adult patients included in the study were hospitalized with the diagnosis of COVID-19 confirmed by SARS-CoV-2 RT-PCR (Abott, USA) after undergoing HSCT, between January 1, 2020, and June 30, 2022. The list of patients was obtained from the Infection Control Unit of the respective hospital. Demographics, clinical and laboratory variables were extracted from patients’ medical records. COVID-19 cases were categorized as asymptomatic, mild, moderate, severe or critical^7^. The primary outcome assessed was all-cause mortality during hospitalization. Additionally, we examined the prevalence of probable invasive aspergillosis as previously defined^8^.

This study was analyzed and approved by the ethics committee of hospital 1 (CAAE Nº 60253222.5.0000.0068) and hospital 2 (CAAE Nº 52240921.0.3003.5455).

### SARS-CoV-2 variants of interest circulating in Brazil

For the purpose of analysis and discussion, we divided the timeline into four periods corresponding to the waves of variants of concern (VOC). This division was based on the results of the Fiocruz Genomic Network, which aims to study SARS-CoV-2 lineages circulating in Brazil^9^:

- Pre-VOC period: from the first case of the cohort until January 31, 2021.
- Gamma period: starting from the first case of the cohort during the circulation of the Gamma variant (February 1, 2021) up to June 30, 2021.
- Delta period: starting from the first case of the cohort during the circulation of the Delta variant (July 1, 2021) up to August 31, 2021.
- Omicron period: starting from the first case of the cohort during the circulation of the Omicron variant (September 1, 2021) up to June 30, 2022

### Sequencing

RNA was extracted using the Extracta 96 kit (Loccus do Brasil Ltda, Sao Paulo, Brazil). Library preparation and sequencing were performed using the COVIDseq kit (Illumina Inc., California, USA) with ARTIC V4.1 primer panel following the manufacture’s recommendations. The quality of the fastq was assessed using FASTQC. Quality reads were aligned to the reference genome (accession Nº NC_045512), variant call, and the generation of the consensus genome sequence was performed with DRAGEN 3.5.13 (Illumina Inc., California, USA). The sublineage analysis was assessed using the Pangolin COVID-19 Lineage Assigner Tool (version 4.0, South Cambridgeshire, UK) and the NextClade online tool (version 3.2.0, Nextstrain, Seattle, WA, USA).

### Statistical analysis

A database was established in Microsoft Excel Microsoft (version 2021, Microsoft, Redmond, WA, USA) and analyzed in Epi InfoTM (version 7.2, CDC, Atlanta, GE, USA). Descriptive analyzes of the clinical variables was performed.

We conducted bivariate and multivariate analyzes to identify variables associated with mortality. Categorical variables were assessed using the Chi-square test or Fisher’s exact test, as appropriate in the bivariate analysis. For variables with levels equal or lower than 0.05 (*p* ≤ 0.05) in bivariate analyses, logistic regression analysis was conducted.

We used forward stepwise modeling for logistic regression. Odds ratios (OR) were calculated along with their respective 95% confidence intervals. A 2-tailed alpha of less than 5% was considered significant for all statistical tests.

## RESULTS

**Figure 1:**
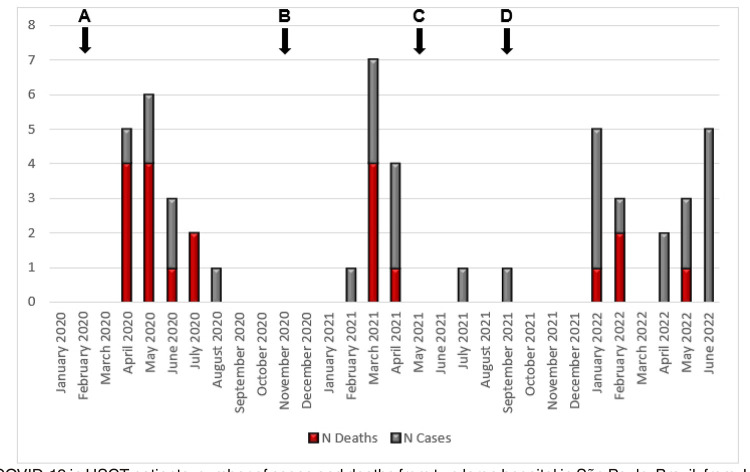
COVID-19 in HSCT patients: number of cases and deaths from two large hospital in São Paulo, Brazil, from January 2020 to June 2022: A) 1st confirmed case of COVID-19 in Brazil (Feb 26, 2020); B) Introduction of the Gamma variant in Brazil (Nov 2020); C) Introduction of the Delta variant in Brazil (May 2021); D) Introduction of the Omicron (BA.1) variant in Brazil (Sep 2021).

A total of 49 cases of COVID-19 acquired after HSCT were included during the study period (28 at Hospital 1, and 21 at Hospital 2). The first COVID-19 case was confirmed in Brazil on February 26, 2020, and the first case of our cohort was identified on April 1, 2020. Figure 1 illustrates the temporal distribution of cases and deaths. The main characteristics of the study population are presented in Table 1.

**Table 1:** Main characteristics of the 49 patients with COVID-19 after HSCT from two large hospital in Sao Paulo, Brazil, January 2020 to June 2022.

	Total (n=49) N (%)
**Hospital 1: n (%)**	28 (57.1)
**Male sex: n (%)**	31 (63.2)
**Age: median (IQR)**	47 (63-33)
**Underlying disease: n (%)**	
Non-Hodgkin’s lymphoma MM AML ALL CML Hodgkin’s lymphoma Other	12 (24.4) 11 (22.4) 7 (14.2) 7 (14.2) 3 (6.1) 3 (6.1) 6 (12.2)
**Type of transplant: n (%)**	
Allogeneic Autologous	25 (51.2) 24 (48.9)
**Charlson’s score: median (min-max)**	2 (0-10)
**Type of conditioning for allogeneic-HSCT: n (%)**	
MAC RIC	18 (75.0) 6 (25.0)
**COVID-19 vaccination status: n (%)**	
No vaccination At least 1 dose At least 2 doses At least 3 doses	35 (71.4) 14 (29.1) 6 (12.5) 5 (10.4)
**Time between HSCT and COVID-19: median days (IQR)**	398 (1,211-134)
**COVID-19 ≤ 12 mo. of HSCT: n (%)**	22 (44.8)
**Source of infection: n (%)**	
Community Nosocomial	38 (77.5) 11 (22.4)
**Disease status at the time of COVID-19 diagnosis: n (%)**	
Complete response Active disease	27 (55.1) 22 (44.8)
**COVID-19 classification: n (%)**	
Asymptomatic Mild Moderate Severe Critical	4 (8.1) 4 (8.1) 9 (18.3) 12 (24.4) 20 (40.8)
**Symptoms at the time of diagnosis: n (%)**	
Fever Cough Upper respiratory symptoms Shortness of breath Headache Fatigue Diarrhea Anosmia Dysgeusia Myalgia	30 (61.2) 23 (46.9) 15 (30.6) 9 (18.3) 5 (10.2) 5 (10.2) 4 (8.1) 3 (6.1) 2 (4.0) 2 (4.0)
**Graft vs. host disease at the time of COVID diagnosis: n (%)**	17 (68.0)
**Under immunosuppression at the time of COVID-19 diagnosis: n (%)**	29 (59.1)
**Laboratory tests at the time of diagnosis: median (IQR)**	
Hb - g/dL Leukocytes/ mm^3^ Lymphocytes/ mm^3^ C-Reactive Protein - mg/dL	9.4 (7.9-11.7) 2,950 (1,840- 6,600) 1,800 (900 - 4,740) 10.1 (5.18-12.9)
**Chest CT involvement at the time of diagnosis: n (%)**	
<25% 25-50% 50-75% >75%	26 (53.0) 6 (12.2) 11 (22.4) 0
**Worst chest CT involvement during follow-up: n (%)**	
<25% 25-50% 50-75% >75%	22 (44.8) 4 (81.6) 14 (28.5) 3 (6.1)
**Use of medication for COVID-19: n (%)** Corticosteroids Remdesivir Convalescent plasma Tocilizumab	33 (67.3) 26 (53.0) 10 (20.4) 6 (12.2) 1 (2.0)
**ICU admission: n (%)**	25 (51.0)
**Supplemental oxygen: n (%)**	32 (65.3)
**Mechanical ventilation: n (%)**	20 (40.8)
**Dialysis: n (%)**	9 (18.3)
**Time of COVID-19 hospitalization (until discharge or death): median days (IQR)**	18 (25-11)
**Mortality: n (%)**	
All-cause mortality COVID-19-related mortality Autologous Allogeneic No vaccination At least 1 dose of vaccine	20 (40.8) 12 (24.4) 11 (45.8) 9 (36.0) 18 (51.4) 2 (14.2)
**Period of diagnosis: n (%)**	
Pre-VOC Gamma Delta Omicron	18 (36.7) 12 (24.4) 1 (2.0) 18 (36.7)

IQR = interquartile range; AML = acute myeloid leukemia; ALL = acute lymphoblastic leukemia; CML = chronic myeloid leukemia; MM = multiple myeloma; MAC = myeloablative conditioning; RIC = reduced intensity conditioning; ^#^Pre-VOC period: from the first case of the cohort until January 31, 2021; Gamma period: first case of the cohort during the circulation of the Gamma variant (February 1, 2021) to June 30, 2021; Delta period: first case of the cohort during the circulation of the Delta variant (July 1, 2021) to August 31, 2021; Omicron period: First case of the cohort during the circulation of the Omicron variant (September 1, 2021) to June 30, 2022.

The majority of patients were male (n=31; 63.2%) with a median age of 47 years old (IQR: 63-33). The main underlying disease was non-Hodgkin’s lymphoma (n=12; 24.4%). Regarding the type of transplant, 25 (51.2%) patients underwent allogeneic-HSCT, while 24 (48.9%) received autologous-HSCT. The median time between HSCT and COVID-19 diagnosis was 398 days (IQR: 1,211-134), with 22 (44.8%) cases occurring within 12 months of transplantation.

Most patients developed fever (61.2%) and cough (46.9%) as the first symptoms of the disease. Only 18.3% had shortness of breath at presentation. A small proportion of patients (8.1%) had gastrointestinal involvement, primarily presenting with diarrhea. Classical symptoms of anosmia and dysgeusia were presented in only 6.1% and 4% of cases, respectively, all of them during the Gamma variant wave. Two patients (4%) had a thromboembolic event.

Seven (14.2%) SARS-CoV-2 cDNA samples were sequenced, and the most frequent VOCs were Gamma and Omicron variant BA.2,with three samples each. The remaining sample belonged to the Omicron variant BA.4. All were detected in their respective VOC period.

Thirty-three (67.3%) patients received some specific medication for COVID-19. The medications prescribed were corticosteroids (n=26; 53.0%), remdesivir (n=10; 20,4%), convalescent plasma (n=6; 12.2%) and tocilizumab (n=1; 2.0%).

### Outcomes

Most patients developed severe (n=12; 24.4%) or critical (n=20; 40.8%) disease. Twenty-five (51%) patients were admitted to the intensive care unit (ICU) and 20 (40.8%) required mechanical ventilation.

All-cause mortality was 40.8% and COVID-19-related mortality was 24.4%. A significant proportion of patients were not vaccinated against COVID-19: 35 (71.4%), but seventeen (34.6%) developed COVID-19 in 2020, when vaccine was not available. Of the latter, 11 (64.7%) died. Remarkably, mortality was 51.2% in non-vaccinated patients and 14.2% in patients who had received at least one dose of the vaccine.

Table 2 shows the bivariate analyses. Age over 50 years old; no COVID-19 vaccination; severe/ critical COVID-19; chest CT involvement at diagnosis ≥50%; corticosteroid use for COVID-19; ICU admission; need for supplemental oxygen and mechanical ventilation were associated with death. COVID-19 diagnosis during the Omicron period was protective. After conducting multiple regression analyses (Supplementary Table S1), we found that mechanical ventilation (OR: 101.01; 95% CI: 8.205 – 1,242.93; *p* = 0.003) and chest CT involvement at diagnosis ≥50% (OR: 26.61; 95% CI: 1.06 – 664.26; *p* = 0.04) were the variables that remained associated with all-cause mortality.

**Table 2:** Variables associated with all-cause mortality in HSCT patients with COVID-19: bivariate analyses.

	Dead n=20 n (%)	Alive n=29 n (%)	RR (95% CI)	*P* value
**Male sex**	11 (55.0)	20 (68.9)	1.29 (0.75 – 2.19)	0.24
**Age** **>** **50 y.**	13 (65.0)	11 (37.9)	1.57 (0.95 – 2.58)	**0.05**
**Type of transplant n (%)**				
Allogeneic Autologous	9 (45.0) 11 (55.0)	16 (55.1) 13 (44.8)	0.84 (0.52 – 1.35) 1.18 (0.77 – 1.89)	0.34 0.34
**N** ^ **o** ^ **COVID-19 vaccination**	18 (90.0)	17 (58.6)	1.76 (1.18-2.63)	**0.01**
**COVID-19 ≤ 12 mo. after HSCT**	8 (40.0)	14 (48.2)	0.87 (0.54 – 1.38)	0.39
**Community acquired infection**	13 (65.0)	25 (86.2)	1.80 (0.80 – 4.08)	0.08
**Active hematological disease at the time of COVID-19 diagnosis**	12 (60.0)	10 (34.4)	1.54 (0.92 – 2.60)	0.07
**Severe/ Critical COVID-19**	19 (95.0)	13 (44.8)	2.31 (1.49 – 3.58)	**0.0001**
**Immunosuppression at the time of COVID-19 diagnosis**	12 (60.0)	17 (58.6)	1.04 (0.64 – 1.68)	0.55
**Chest CT involvement** ≥ **50% at the time of diagnosis**	11 (55.0)	1 (3.4)	8.25 (1.26 – 53.97)	**0.0001**
**Use of corticosteroids for COVID-19**	14 (70.0)	12 (41.3)	1.60 (0.99 – 2.59)	**0.04**
**ICU admission**	19 (95.0)	6 (12.2)	3.99 (1.97 – 8.06)	**< 0.0001**
**Mechanical ventilation**	18 (90.0)	2 (6.8)	9.31 (2.49 – 34.80)	**<0.0002**
**Period of diagnosis**				
Pre-VOC Gamma Delta **Omicron**	11 (55.0) 5 (25.0) 0 4 (20.0)	6 (20.6) 7 (24.1) 1 (3.4) 15 (51.7)	1.82 (0.98 – 3.39) 1.01 (0.58 – 1.76) 0.58 (0.45 – 0.74) 0.62 (0.40- 0.96)	0.02 0.60 0.59 **0.04**

Pre-VOC period:from the first case of the cohort until January 31, 2021; Gamma period: first case of the cohort during the circulation of the Gamma variant (February 1, 2021) to June 30, 2021; Delta period: first case of the cohort during the circulation of the Delta variant (July 1, 2021) to August 31, 2021; Omicron period: first case of the cohort during the circulation of the Omicron variant (September 1, 2021) to June 30, 2022.

The prevalence of probable invasive aspergillosis was 10.2% (n=5). Galactomannan concentrations in bronchoalveolar lavage varied from 1.0 to 5.46 ng/mL. Two cases of COVID-19 after autologous-HSCT and three cases after allogeneic-HSCT resulted in probable invasive aspergillosis prevalences of 8.3% and 25.0%, respectively. Among these cases, two (20.0%) patients died, both of whom had undergone autologous transplantation. One of them developed a brain abscess and exhibited a serum galactomannan of 7.23 ng/mL. Even though the exact etiology of the abscess remained unidentified, we postulated that a potential cause could be a brain aspergilloma. The characteristics of probable cases of invasive aspergillosis cases are summarized in the Supplementary Table S2.

## DISCUSSION

Our study observed a high mortality rate among hospitalized HSCT patients diagnosed with COVID-19. Classical symptoms as anosmia and dysgeusia were not frequent and were reported only during the Gamma variant wave. The most commonly detected VOC among the sequenced samples was Omicron BA.2. Mechanical ventilation and chest CT involvement ≥50% at diagnosis were independent risk factors associated with death.

COVID-19 vaccination was launched on January 18, 2021, in Brazil, initially prioritizing individuals over 90 years of age. Starting on May 10, 2021, immunocompromised patients of any age, including HSCT recipients, became eligible for vaccination. The COVID-19 vaccines administered in Brazil were CoronaVac^®^, ChAdOx1 nCov-19 (Oxford-AstraZeneca^®^), Ad26.COV2.S (Janssen^®^) and BNT162b2 mRNA (Pfizer-BioNTech^®^)^10^. In the bivariate model of our study, the lack of COVID-19 vaccination was associated with mortality. It is possible that this variable was not significant in the multiple regression analyses because of the relatively small number of patients in our cohort.

Notably, another Brazilian study involving 313 healthcare workers with confirmed COVID-19 attributed to the Gamma variant observed a remarkable prevalence of the classical symptoms of anosmia (n=78; 25.0%) and dysgeusia (n=66; 21.0%)^11^. The relatively limited occurrence of these symptoms in our cohort were not expected.

A recent meta-analysis observed a pooled mortality rate of 17% of COVID-19 cases following HSCT^5^, a significant lower percentage when compared to the rate reported by us. This discrepancy could be attributed, in part, to the fact that our study exclusively included hospitalized patients. Nevertheless, the mortality of COVID-19 in the HSCT population has been previously reported to be as high as 42.1%^12^, which is similar to our findings.

To the best of our knowledge, this study is the first among COVID-19 HSCT patients to demonstrate an independent association between mechanical ventilation and chest CT involvement at diagnosis with death^1,4,6,13-19^. In another meta-analysis, a subgroup analysis revealed a higher risk of death among patients who develop COVID-19 within 12 months of HSCT and in those with GVHD using immunosuppressant drugs^3^.

The prevalence of probable aspergillosis was 10.2%. Notably, two of the five cases (40.0%) of probable aspergillosis were diagnosed in autologous HSCT, both of whom died. We hypothesize that COVID-19 may have increased the risk of invasive fungal infection in these patients. Limited studies have investigated the incidence of aspergillosis in hematological patients with COVID-19. In a retrospective study involving 41 patients with AML, 5 (12.0%) developed probable aspergillosis, and one (20%) of them died^20^.

Our study has certain limitations. It is based on a relatively small retrospective cohort of HSCT patients diagnosed with COVID-19. Additionally, genome sequencing was not available for all patient samples. Considering that the majority of our cases occurred during the pre-vaccination period, it is not recommended that the data presented here be directly compared with current ones obtained in era of widespread vaccination. Finally, the small sample size prevented us from analyzing outcomes in subgroups, such as the diagnostic period in relation to VOC. Nonetheless, our study encompassed patients from two large hospitals in Brazil during three years of the pandemic, covering the period before and after the introduction of COVID-19 vaccination.

## CONCLUSION

In conclusion, we presented a cohort of HSCT patients with COVID-19 since the beginning of the pandemic. The prevalence of probable aspergillosis after COVID-19 was not negligible, even after autologous HSCT. A high mortality rate was observed after COVID-19, and the need of mechanical ventilation as well as chest CT involvement ≥ 50% at diagnosis were associated with death. Thus, it is essential to fully vaccinate the population and implement measures to avoid the risk of SARS-CoV-2 infection.
